# Effects of Aprepitant on the Pharmacokinetics of Controlled-Release Oral Oxycodone in Cancer Patients

**DOI:** 10.1371/journal.pone.0104215

**Published:** 2014-08-14

**Authors:** Yutaka Fujiwara, Masanori Toyoda, Naoko Chayahara, Naomi Kiyota, Takanobu Shimada, Yoshinori Imamura, Toru Mukohara, Hironobu Minami

**Affiliations:** 1 Division of Medical Oncology/Hematology, Kobe University Graduate School of Medicine, Kobe, Japan; 2 Cancer Center, Kobe University Graduate School of Medicine, Kobe, Japan; 3 Division of Investigational Cancer Therapeutics, Exploratory Oncology Research & Clinical Trial Center, National Cancer Center Hospital, Chiba, Japan; George Mason University, United States of America

## Abstract

**Purpose:**

Oxycodone is a µ-opioid receptor agonist widely used in the treatment of cancer pain. The predominant metabolic pathway of oxycodone is CYP3A4-mediated N-demethylation to noroxycodone, while a minor proportion undergoes 3-O-demethylation to oxymorphone by CYP2D6. The aim of this study was to investigate the effects of the mild CYP3A4 inhibitor aprepitant on the pharmacokinetics of orally administered controlled-release (CR) oxycodone.

**Method:**

This study design was an open-label, single-sequence with two phases in cancer patients with pain who continued to be administered orally with multiple doses of CR oxycodone every 8 or 12 hours. Plasma concentration of oxycodone and its metabolites were measured up to 8 hours after administration as follows: on day 1, CR oxycodone was administered alone; on day 2, CR oxycodone was administered with aprepitant (125 mg, at the same time of oxycodone dosing in the morning). The steady-state trough concentrations (Css) were measured from day 1 to day 3.

**Results:**

Aprepitant increased the area under the plasma concentration-time curve (AUC_0–8_) of oxycodone by 25% (p<0.001) and of oxymorphone by 34% (p<0.001), as well as decreased the AUC_0–8_ of noroxycodone by 14% (p<0.001). Moreover, aprepitant increased Css of oxycodone by 57% (p = 0.001) and of oxymorphone by 36% (p<0.001) and decreased Css of noroxycodone by 24% (p = 0.02) at day 3 compared to day 1.

**Conclusions:**

The clinical use of aprepitant in patients receiving multiple doses of CR oxycodone for cancer pain significantly altered plasma concentration levels, but would not appear to need modification of the CR oxycodone dose.

**Trial Registration:**

UMIN.ac.jp UMIN000003580.

## Introduction

Oxycodone is a µ-opioid receptor agonist which is widely used in the treatment of cancer pain and chronic pain [1]. It is a semi-synthetic form of morphine with similar analgesic properties and side effects such as nausea, vomiting, constipation, somnolence, dizziness and pruritus [2]. At high dose or overdoses, oxycodone can cause shallow respiratory depression, somnolence progressing to stupor or coma, skeletal muscle flaccidity, etc. The oral bioavailability of oxycodone is 60 to 87%, and is higher than that of morphine [3]–[5]. Only 10% of the oxycodone dose is excreted unchanged in the urine and it is extensively metabolized by duodenal and hepatic cytochrome P450 (CYP) isozymes [6] [7]. The predominant metabolic pathway of oxycodone is CYP3A4-mediated N-demethylation to noroxycodone, while a minor proportion undergoes 3-O-demethylation CYP2D6 to oxymorphone, which is the active metabolite. Further oxidation of these metabolites via CYP2D6 (and CYP3A4) yields noroxymorphine [6]. Both of these metabolites are further metabolized into noroxymorphine.

Aprepitant, an orally available, selective neurokinin-1 receptor agonist, is effective for both acute and delayed chemotherapy-induced nausea and vomiting (CINV) and is used in combination with a 5-hydroxytryptamine-3 (5HT_3_) antagonist and a corticosteroid (e.g., dexamethasone) for the treatment of moderately and highly emetogenic chemotherapy. The recommended dose of aprepitant is 125 mg prior to chemotherapy on day 1 and 80 mg once daily on days 2 and 3 (125-mg/80-mg regimen).

Aprepitant is metabolized by CYP isozymes 1A2, 2C19, and 3A4, and was shown to be a moderate inhibitor of CYP3A4 (K_i_ of about 10 µM for 1′ and 4-hydroxylation of midazolam and N-demethylation of diltiazem, respectively) in vitro and a very weak inhibitor of CYP2C19 and CYP2C9 [8]. Moreover, drug-drug interaction studies have indicated that aprepitant can inhibit CYP3A4 enzyme activity. When the standard oral dexamethasone regimen for CINV (20 mg on day 1 and 8 mg on days 2 to 5) was given concomitantly with aprepitant, the dexamethasone area under the time-concentration curve (AUC) from 0 to 24 hours increased approximately 2-fold on both day 1 and day 5 compared with the standard oral dexamethasone regimen alone [9]. When the methylprednisolone regimen consisted of 125 mg intravenously on day 1 and 40 mg orally on days 2 to 3, aprepitant increased the AUC of intravenous methylprednisolone 1.3-fold on day 1 and of oral methylprednisolone 2.5-fold on day 3 [9]. Conversely, several studies have not demonstrated that aprepitant use mediated clinically relevant effects on the pharmacokinetics of intravenously administered docetaxel or vinorelbine [10] [11].

At the 125-mg/80-mg regimen used for oral aprepitant administration for CINV, the peak plasma concentrations (Cmax) of 1,600 ng/mL (around 3.0 µM) and 1,400 ng/mL (around 2.6 µM) were reached in approximately 4 hours (Tmax) on day 1 and day 3, respectively [12]. As the intestinal drug concentration following oral administration is even higher than the plasma concentration, it is expected that orally-administered aprepitant inhibits intestinal CYP3A4 greater than intravenously-administered aprepitant and that orally co-administered drug is affected to a greater extent by the inhibitory effect of intestinal CYP3A4 than intravenously co-administered drug [9], [13].

Concomitant use of oxycodone and aprepitant is used in clinical practice for cancer patient care. However, aprepitant might have the potential to increase the plasma concentrations of oxycodone and its metabolites via inhibition of CYP3A-mediated metabolism of oxycodone. As a result, the side effects of oxycodone may increase. In this study, we have therefore investigated the possible effects of the mild CYP3A4 inhibitor aprepitant on the pharmacokinetics of orally administered CR oxycodone in patients with cancer pain.

## Methods

The protocol for this trial and supporting TREND checklist are available as supporting information; see Checklist S1, Protocol S1 and S2.

### Patient selection criteria

The subjects were enrolled in patients whom continued to be administered CR oxycodone twice or three times daily for cancer pain and were planned to receive chemotherapy with aprepitant for CINV. Within the last 3 or more days to reach steady state, the subjects had received a fixed dose of CR oxycodone. Additional eligibility criteria were age≥18 years, histologically confirmed malignant solid tumor, and adequate organ function [serum total bilirubin less than 1.5×upper limits of normal (ULN), aspartate aminotransferase (AST) less than 2.5×ULN, alanine aminotransferase (ALT) less than 2.5×ULN, and serum creatinine less than 1.5×ULN]. Patients were excluded if they had gastrointestinal disorders that could affect ingestion or absorption of either CR oxycodone or aprepitant, and if they were receiving or likely to receive drugs or food that could act as potent CYP3A4 or CYP2D6 inhibitors or inducers. All patients provided written informed consent and study approval was obtained from the Institutional Review Board of Kobe University Hospital.

### Study design

This study which was an open-label, two-period, single-sequence design was conducted at Kobe University Hospital. Patients were administered regularly with multiple-doses of oral CR oxycodone every 8 or 12 hours. Each patient was administered with the appropriate dose of oral CR oxycodone for their cancer pain. They received CR oxycodone alone (period A) on the previous day of planned chemotherapy and CR oxycodone with aprepitant (period B) on the day of chemotherapy. On the morning of period B, aprepitant was taken orally at the same time as CR oxycodone more than one hour prior to chemotherapy. Patients were participated in this study during blood sampling. Patients in hospital were given the dose of anticancer agents according to standard treatment schedule for their tumor types and were allowed to receive an antiemetic treatment with dexamethasone and a 5HT_3_ receptor antagonist where appropriate.

### Outcome

The study objective was to investigate aprepitant might have the potential to increase the plasma concentrations of oxycodone and its metabolites via inhibition of CYP3A-mediated metabolism of oxycodone. The primary endpoint was pharmacokinetics of oxycodone and its metabolites with and without aprepitant administration. Secondary endpoints were safety and adverse event including nausea, vomiting, constipation, and somnolence. Patient characteristics and medication information were recorded throughout the study. Adverse events were evaluated using the CTCAE v4.0.

### Blood sampling

Blood samples for pharmacokinetic analysis were collected immediately before and 1, 2, 3, 5, and 8 hour after administration of oxycodone in periods A and B. An additional sample was collected to allow for analysis of trough concentration before administration of oxycodone in the morning on the following day of period B. After blood was collected in lithium heparin-containing tubes, plasma was separated within 30 min by centrifugation at 1,500×g for 10 min at 4°C and stored at −80°C until analysis. Plasma concentrations of oxycodone, noroxycodone, and oxymorphone were determined using a liquid chromatography tandem mass spectrometric method. The lower limit of quantification was 0.1 ng/ml.

### Pharmacokinetic analysis

Pharmacokinetic variables of oxycodone, noroxycodone, and oxymorphone were determined using the Pheoenix WinNonlin pharmacokinetic program (Pharsight, Mountain View, California). The Cmax and time to maximum concentration (Tmax) were observed directly from the data. The AUC with extrapolation to 8 hour (AUC_0–8_) was calculated by the trapezoidal rule. The linear trapezoidal rule was used for successive increasing concentration values, and the logarithmic trapezoidal rule for decreasing concentration values. Metabolite-to-parent drug AUC ratios (AUCm/AUCp) were calculated to compare the relative abundance of each metabolite.

### Statistical Analysis

This study was designed in order to exclude a clinically significant higher exposure to oxycodone and its metabolites. The null hypothesis was that coadministration of aprepitant would not increase the plasma concentration of oxycodone to a clinically meaningful degree, i.e., the ratio of the geometric mean AUC_0→8_ for oxycodone between period A and period B would be <1.33. Package insert of oxycodone reports that the AUC of oxycodone in steady state was 216.2±97.4 ng.hr/ml [mean ± standard deviation, coefficient of variance (CV) was 45.1%] in patients with cancer pain (n = 32). We estimated that 20 subjects were needed to detect a 33% difference in the AUC_0→8_ for oxycodone at a power of 80% and level of significance p<0.05 (two-sided). The calculations used the sample size procedures in NCSS PASS 11 software. Data are expressed as the geometric mean ± SD. Statistical significance of logarithmic geometric means in AUC and Cmax was analyzed using a paired Student’s t-test, with a probability level of 0.05 used as the criterion of significance. Tmax was analyzed using a Wilcoxon signed-rank test. All statistical analyses were performed with NCSS 2007 (NCSS, LLC. Kaysville, UT).

## Results

### Patient population

**Figure 1:**
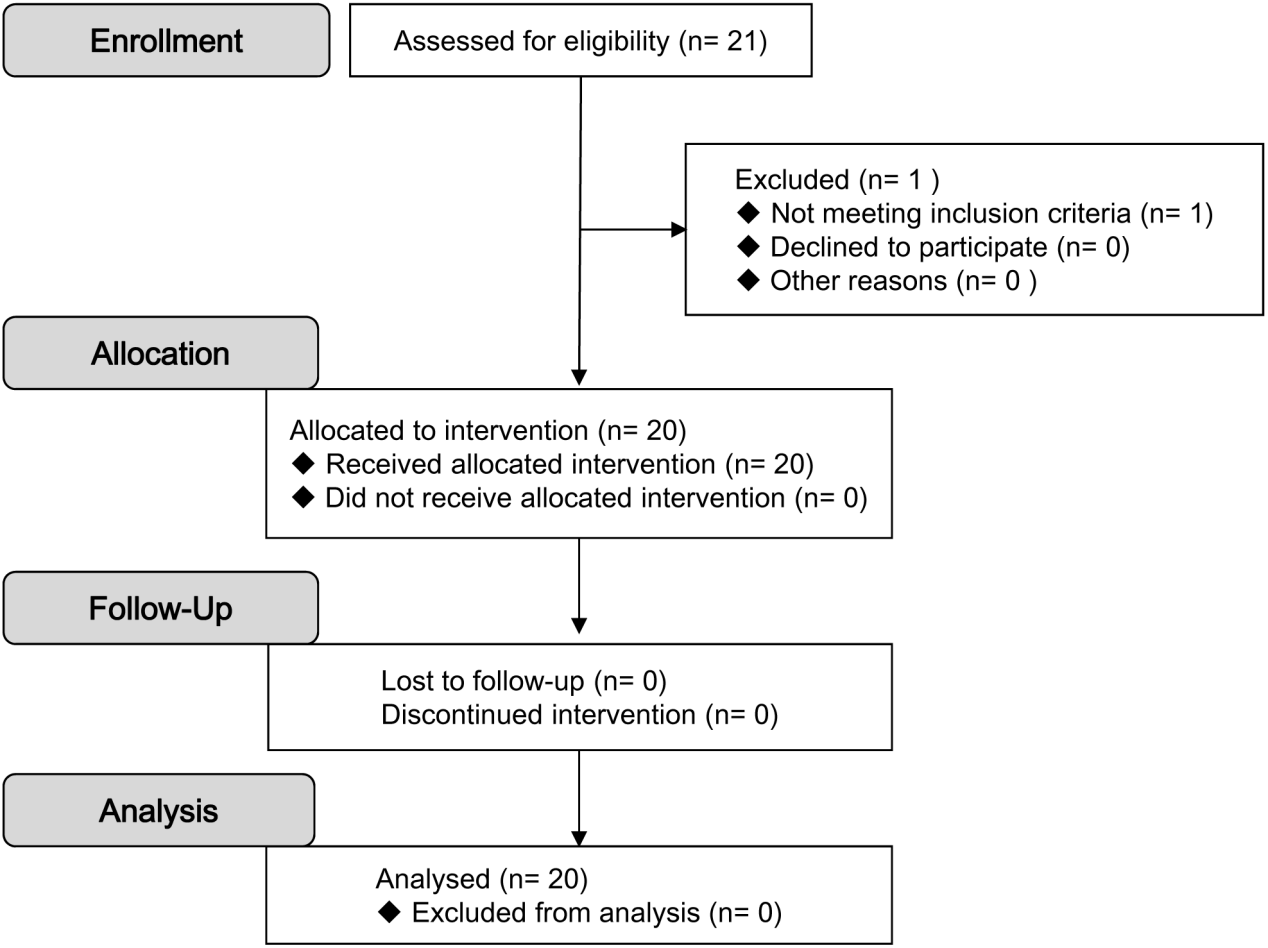
CONSORT flow diagram.

Twenty one patients were assessed for eligibility and 20 patients were allocated to intervention from September 2010 to December 2012 (Figure 1). Their characteristics are listed in Table 1. There were 17 men and 3 women with Eastern Cooperative Oncology Group performance status 1 to 2. The predominant tumor types were pancreatic cancer and head and neck cancer, with all patients having stage IV disease. Each patient was administered regularly with the appropriate dose of oral CR oxycodone every 8 or 12 hours (Table 2). The median daily dosage of oxycodone was 20 mg (range, 10–60 mg) and the mean was 21.5 mg, with the median for each dosage being 10 mg (range, 5–20 mg) and the mean being 9.25 mg.

**Table 1:** Patient characteristics.

		Number of Patients (n = 20)
Gender	Male/female	17 (85%)/3 (15%)
Age	Median (range)	66.5 (44–77)
ECOG PS	1/2	13 (65%)/7 (35%)
Height (cm)	Median (range)	164.4 (138.5–177.1)
Weight (kg)	Median (range)	59.6 (37–77)
BSA (m^2^)	Median (range)	1.64 (1.19–1.90)
Cancer type	Pancreatic cancer	8 (40%)
	Head and Neck cancer	4 (20%)
	NSCLC	2 (10%)
	CRC	2 (10%)
	CUP	2 (10%)
	Endometrial cancer	1 (5%)
	Cholangiocarcinoma	1 (5%)
Clinical stage	IV	20 (100%)
Anti-cancer agent	Platinum agent	8 (40%)
	Gemcitabine	7 (35%)
	Fluoropyrimidine	5 (25%)
	Taxanes	4 (20%)
	Anthracyclines	2 (10%)
	Irinotecan	2 (10%)
	Sunitinib	1 (5%)

Abbreviations: ECOG PS, Eastern Cooperative Oncology Group Performance Status; NSCLC, non-small cell lung cancer; CRC, colorectal cancer; CUP, cancer of unknown primary.

**Table 2:** Dose of Oxycodone.

Dose	Frequency of administration	Daily dosage	Number of patients
5 mg	every 12 hours	10 mg	6 (30%)
	every 8 hours	15 mg	2 (10%)
10 mg	every 12 hours	20 mg	6 (30%)
	every 8 hours	30 mg	3 (15%)
15 mg	every 12 hours	30 mg	1 (5%)
20 mg	every 12 hours	40 mg	1 (5%)
	every 8 hours	60 mg	1 (5%)

### Oxycodone and its metabolites pharmacokinetics

**Figure 2:**
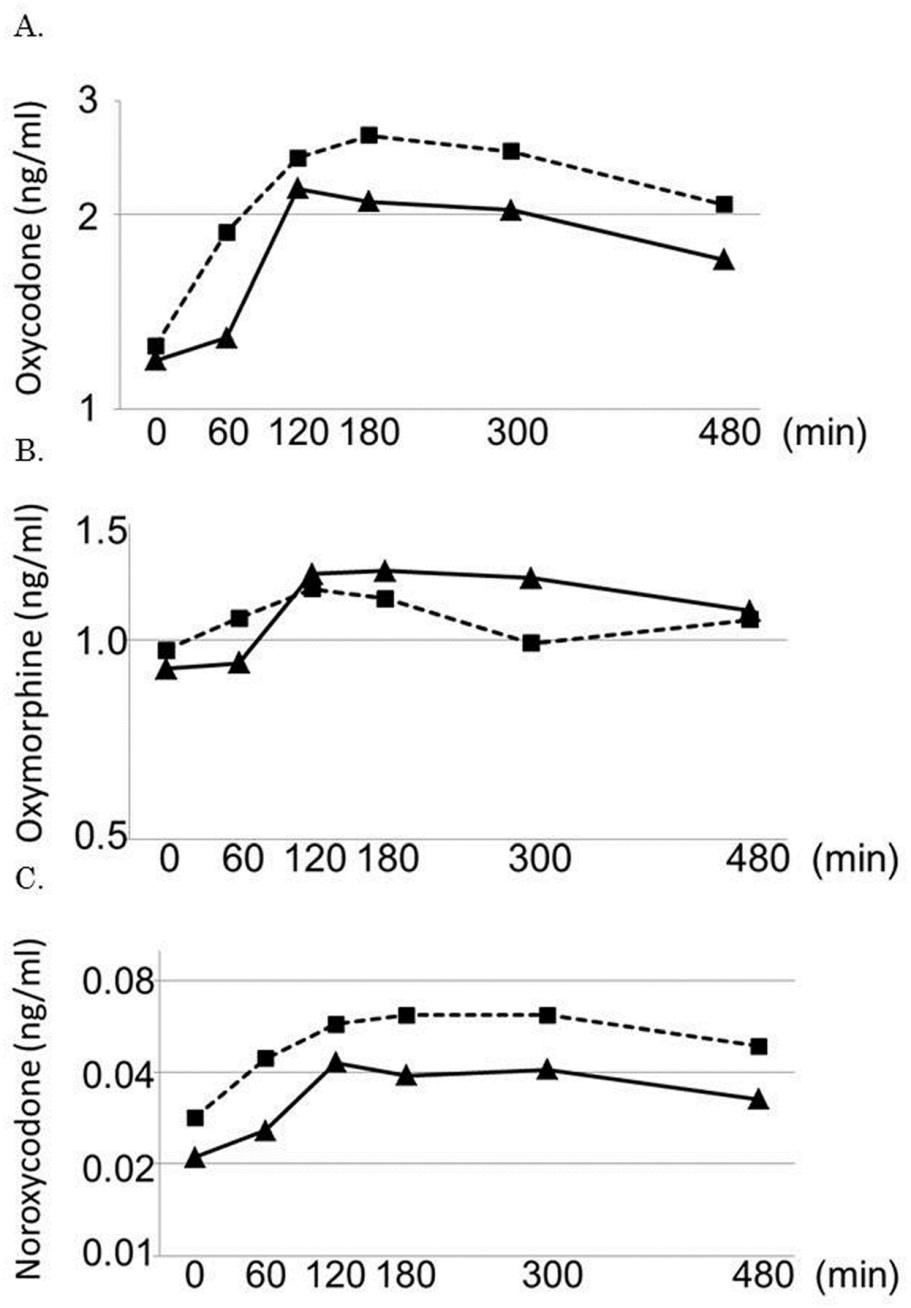
Mean plasma concentration curves of oxycodone, noroxycodone, and oxymorphone in patients (n = 6) who were administered with 10 mg of CR oxycodone every 12 hours alone (period A, triangles) or with aprepitant (period B, squares). ▴ without aprepitant, ▪ with aprepitant.

All 20 patients were assessed for pharmacokinetics of oxycodone and its metabolites with and without aprepitant administration. In five patients who were administered with 5 mg of oral CR oxycodone every 12 hours, the plasma oxymorphone concentration was below the limit of quantification. Table 3 and 4 summarize the pharmacokinetic parameters of oxycodone administered alone or with aprepitant. Figure 2 shows the geometric mean plasma concentrations of oxycodone and its metabolites in patients (n = 6) who were administered with 10 mg of CR oxycodone every 12 hours alone or with aprepitant. The ratio of the geometric mean AUC_0–8_ and Cmax of CR oxycodone plus aprepitant [1,102 ng*hr/ml (CV 29.9%) and 2.79 ng/ml (CV 28.0%), respectively] to those of CR oxycodone alone [882 ng*hr/ml (CV 35.7%) and 2.28 ng/ml (CV 31.4%), respectively] was 1.25 (95% CI 1.14, 1.36; CV 21.8%; p = 0.00004) and 1.22 (95% CI 1.11, 1.34; CV 20.6%; p = 0.0002), respectively. The ratio of the geometric mean AUC_0–8_ of noroxycodone and oxymorphone with aprepitant [616 ng*hr/ml (CV 51.6%) and 20.7 ng*hr/ml (CV 65.8%), respectively] to those without aprepitant [718 ng*hr/ml (CV 45.2%) and 14.9 ng*hr/ml (CV 78.0%), respectively] was 0.86 (95% CI 0.81, 0.91; p = 0.00005) and 1.34 (95% CI 1.20, 1.49; p = 0.00004), respectively. The plasma concentrations of oxycodone and its metabolites were affected significantly by presence or absence of aprepitant.

**Table 3:** Pharmacokinetic parameter of oxycodone and its metabolites.

	Oxycodone			Noroxycodone	Oxymorphone
	Cmax (ng/mL)	Tmax (hr)	AUC_0→8_(ng*hr/mL)	AUC_0→8_(ng*hr/mL)	AUC_0→8_(ng*hr/mL)
Number of patients	20	20	20	20	15**
Without aprepitant	2.28	2.67	882	718	14.9
	(31.4%)	(57.7%)	(35.7%)	(45.2%)	(78.0%)
With aprepitant	2.79	3.62	1102	616	20.7
	(28.0%)	(32.1%)	(29.9%)	(51.6%)	(65.8%)
ratio	1.22		1.25	0.86	1.34
	(1.11–1.34)		(1.14–1.36)	(0.81–0.91)	(1.20–1.49)
p-value*	0.0002	0.07	0.00004	0.00005	0.00004

Abbreviations: Cmax, peak plasma concentration; Tmax, time to peak plasma concentration; AUC_0→8_, area under the time-concentration curve from 0 to 8 hours; ratio, the ratio of the geometric mean value of CR oxycodone with aprepitant to those without aprepitant.

Geometric mean (% coefficient variance).

Values were corrected for dose, assuming that all patients received 20 mg of oxycodone.

*Paired t-test for difference between logarithmic geometric means (two-sided).

**Five patients were excluded due to below lower limit of quantitation.

**Table 4:** Trough concentrations of oxycodone and its metabolites.

	Oxycodone		Noroxycodone		Oxymorphone	
	N	(ng/mL)	N	(ng/mL)	N	(ng/mL)
Day 1 pre-dose	20	1.29	20	1.28	14	0.0243
Without aprepitant		(53.1%)		(46.2%)		(72.7%)
Day 2 pre-dose	20	1.22	20	1.23	14	0.0277
Without aprepitant		(49.3%)		(47.8%)		(68.8%)
Day 3 pre-dose	19	2.00	19	0.97	17	0.0321
With aprepitant		(49.2%)		(54.5%)		(78.8%)
Ratio (D3 to D1)	19	1.57	19	0.760	13	1.36
p-value*		0.001		0.00003		0.02
Ratio (D3 to D2)	19	1.65	19	0.796	13	1.32
p-value*		0.0001		0.00001		0.02

Abbreviations: N, number of patients; Ratio (D3 to D1), the ratio of the geometric mean trough concentration of CR oxycodone plus aprepitant on day 3 to those of CR oxycodone alone on day 1; Ratio (D3 to D2), the ratio of the geometric mean trough concentration of CR oxycodone plus aprepitant on day 3 to those of CR oxycodone alone on day 2.

Geometric mean (% coefficient variance).

Values were corrected for dose, assuming that all patients received 20 mg of oxycodone.

*Paired t-test for difference between logarithmic geometric means (two-sided).

The trough concentration of oxycodone and its metabolite on day 1 were similar to those on day 2, because steady state was reached. However, these trough concentrations with aprepitant on day 3 were higher than those on day 1 and day 2. The ratio of the geometric mean trough concentration of CR oxycodone plus aprepitant on day 3 to those of CR oxycodone alone on day 2 was 1.65 in oxycodone (p = 0.0001), 0.796 in noroxycodone (p = 0.00001), and 1.32 in oxymorphone (p = 0.02), respectively.

In this study and clinical practice, there was no increased incidence in pharmacologic effect and side effects of oxycodone due to concomitant use of aprepitant.

## Discussion

**Figure 3:**
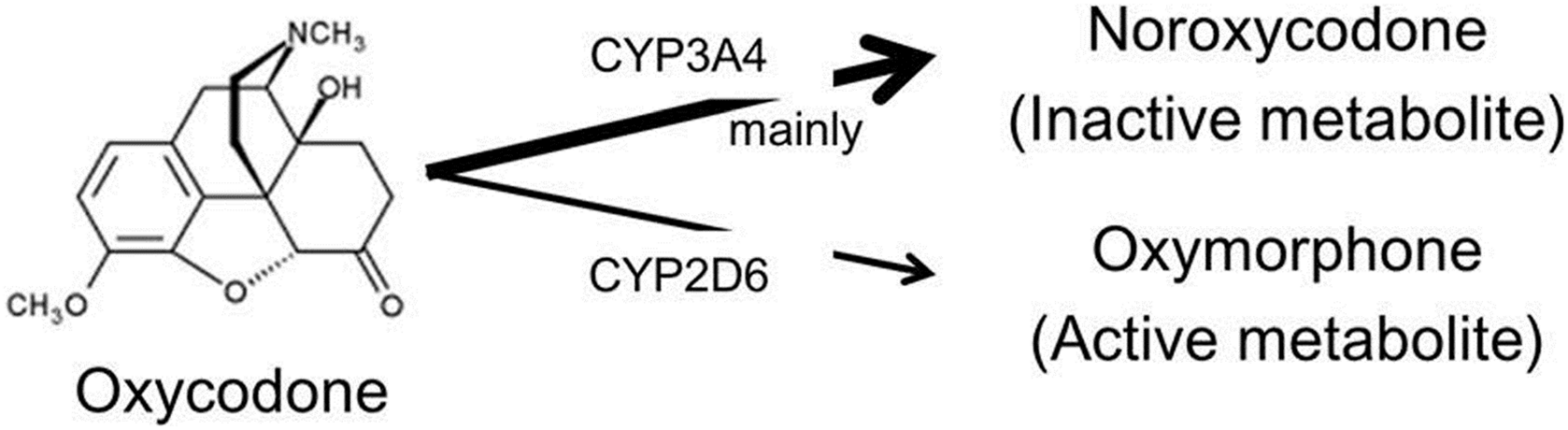
Metabolic pathway of Oxycodone.

The predominant metabolic pathway of oxycodone is CYP3A4-mediated N-demethylation to noroxycodone, while a minor proportion undergoes 3-O-demethylation to oxymorphone by CYP2D6 (Figure 3). This study demonstrated that inhibition of CYP3A4-mediated N-demethylation by aprepitant significantly increased the AUC of oxycodone by 25% and decreased the AUC of noroxycodone by 14%, while subsequently increasing the AUC of oxymorphone by 34% through alternating CYP2D6 pathway. We estimated in advance that a clinical meaningful significant level of interaction between oxycodone and aprepitant would be a 33% increase in the ratio of the geometric mean AUC_0→8_ under conditions where the CV was 45.1%. Essentially, the impact of aprepitant upon oxycodone was less than expected but the actual CV in the AUC of oxycodone was 30 and 35% in this study. Therefore, we consider that statistical significance was achieved as a result. In this study and clinical practice, there would be no increased incidence in pharmacologic effect and side effects of oxycodone due to concomitant use of aprepitant. We consider that a 25% increase (median 1.25; 95% CI 1.14, 1.36) in the ratio of the geometric mean AUC_0→8_ is a statistically significant effect, but that, due to its less extent than expected, at this time there is no need to change the CR oxycodone dose in clinical use of aprepitant in cancer patients, with adequate attention. With regard to oxymorphone which is an active metabolite, because oxymorphone is a potent opioid that has a 4 to 6 times lower µ-opioid receptor affinity and lower concentration than oxycodone [6] [14], an increase of oxymorphone would be unlikely to have a significant impact on the clinical relevance. However, because the recommended dose of aprepitant is 125-mg/80-mg regimen over 3 days, it is important to further investigate the possible effects of the 125-mg/80-mg aprepitant regimen on the pharmacokinetics of orally administered CR oxycodone in patients with cancer pain.

Aprepitant had no detectable inhibitory effect on the pharmacokinetics of intravenously administered docetaxel or vinorelbine [10] [11] but resulted in increased plasma concentration of orally administered dexamethasone or CR oxycodone [9]. It is expected that an orally-coadministered drug is affected to a greater extent by an inhibitory effect of intestinal CYP3A4 than intravenously-administered drug due to the higher intestinal concentration of aprepitant as compared to the plasma concentration. Therefore, we consider that this result for CR oxycodone may not be applicable to intravenously administered oxycodone. In this study, our patients received individual dose and schedule of CR oxycodone and combined with various anti-cancer agents according to standard treatment for their tumor types. Additionally, we didn’t conducted placebo-controlled trial, because the primary endpoint in this study is not pharmacodynamics of oxycodone and its metabolites but pharmacokinetics. These are limitations of study, because this study was conducted in subjects whom continued to be administered CR oxycodone routinely for cancer pain. Further study to validate effects of aprepitant on the pharmacokinetics and pharmacodynamics of controlled-release oral oxycodone pharmacokinetic is expected.

The trough concentration of oxycodone and its metabolite on day 1 pre-dose were similar to those on day 2 pre-dose, despite these trough concentrations with aprepitant on day 3 were higher than those on day1 and day 2. This indicated that the trough concentrations of CR oxycodone alone at steady state were not observed inter-day variability (Table 4). Meanwhile, the ratio of the geometric mean AUC_0–8_ and trough concentration of CR oxycodone plus aprepitant to those of CR oxycodone alone was 1.25 (range 0.98–1.96) and 1.65 (range 0.54–3.41), respectively, with wide inter-patient variability observed (Figures S1 and S2). A pharmacogenomics study showed that a CYP2D6 genotype had an impact on plasma concentrations of oxycodone and oxymorphone, and the metabolism of oxycodone [15]. First, we are now planning a further pharmacogenomics study. Secondly, we will analyze plasma concentrations of aprepitant and investigate the possible influence of aprepitant concentrations on the pharmacokinetics of orally administered CR oxycodone.

In conclusion, aprepitant increased the exposure of oxycodone by 25% due to inhibiting its CYP3A4-mediated N-demethylation. The clinical use of aprepitant in patients receiving multiple doses of CR oxycodone for cancer pain significantly altered plasma concentration levels, but would not appear to need modification of the CR oxycodone dose in clinical co-administration of aprepitant in cancer patients, with adequate attention.

## Supporting Information

Figure S1 **Individual value plot of AUC_0–8_ of (A) oxycodone (n = 20), (B) noroxycodone (n = 20), and (C) oxymorphone (n = 15) in patients who were administered with CR oxycodone alone or with aprepitant.** Dose of CR oxycodone: circle (5 mg), triangle (10 mg), square (15 mg), and pentagon (20 mg).
(TIF)

Figure S2 **Individual value plot of trough concentration of (a) oxycodone (n = 19), (b) noroxycodone (n = 19), and (c) oxymorphone (n = 13) in patients who were administered with CR oxycodone alone or with aprepitant.** Dose of CR oxycodone: circle (5 mg), triangle (10 mg), square (15 mg), and pentagon (20 mg).
(TIF)

Checklist S1 **TREND Statement Checklist.**
(PDF)

Protocol S1 **Clinical Study Protocol (Japanese version).**
(PDF)

Protocol S2 **Clinical Study Protocol (English version).**
(DOCX)
