# Supplementary figures and images for: Effects of Aprepitant on the Pharmacokinetics of Controlled-Release Oral Oxycodone in Cancer Patients
Source: PLoS One. 2014 Aug 14;9(8):e104215. doi: 10.1371/journal.pone.0104215 (PMC4133207; doi:10.1371/journal.pone.0104215)

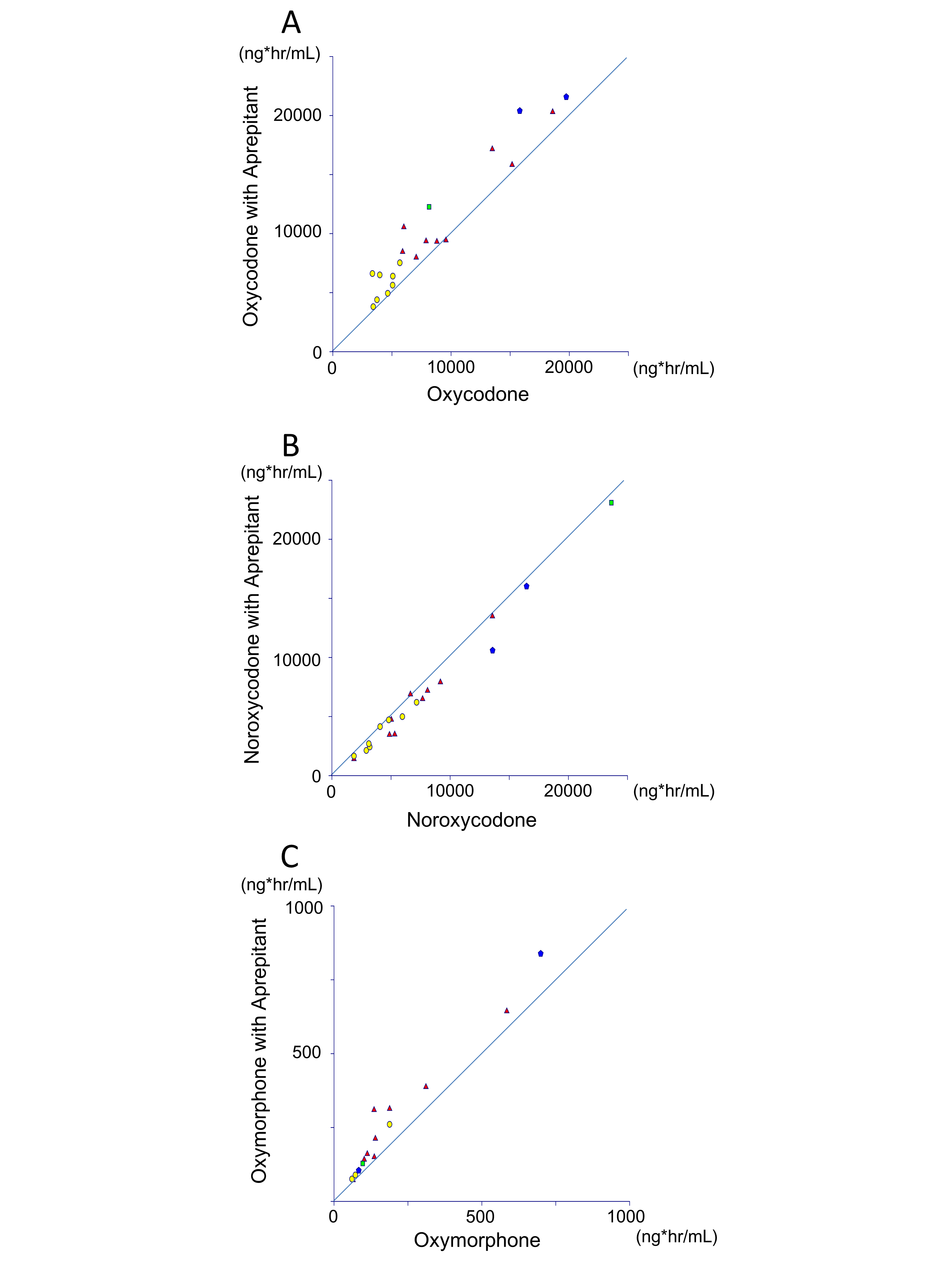

Supplement: Figure S1 — Individual value plot of AUC0–8 of (A) oxycodone (n = 20), (B) noroxycodone (n = 20), and (C) oxymorphone (n = 15) in patients who were administered with CR oxycodone alone or with aprepitant. Dose of CR oxycodone: circle (5 mg), triangle (10 mg), square (15 mg), and pentagon (20 mg). (TIF) [file pone.0104215.s001.tif]

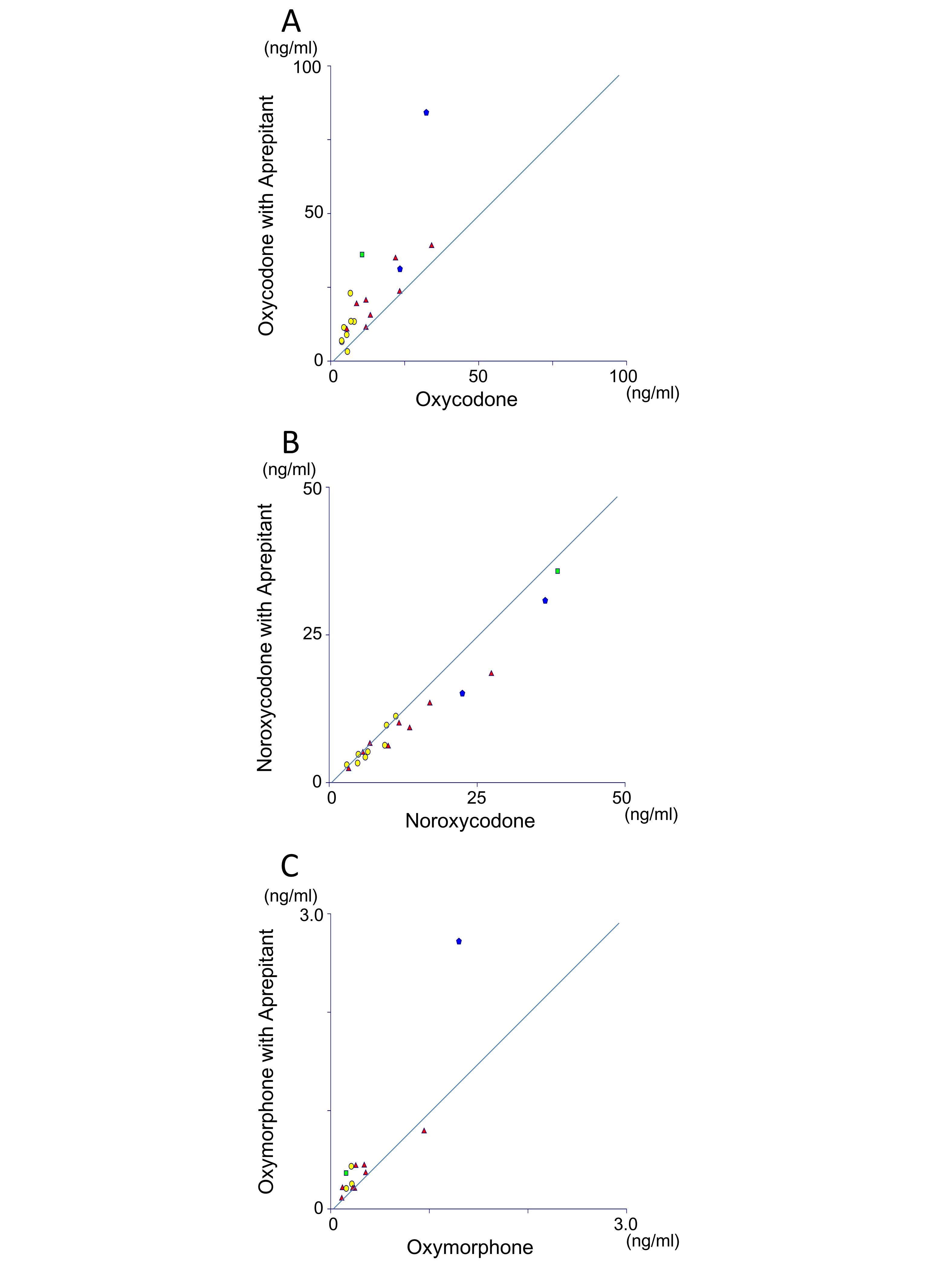

Supplement: Figure S2 — Individual value plot of trough concentration of (a) oxycodone (n = 19), (b) noroxycodone (n = 19), and (c) oxymorphone (n = 13) in patients who were administered with CR oxycodone alone or with aprepitant. Dose of CR oxycodone: circle (5 mg), triangle (10 mg), square (15 mg), and pentagon (20 mg). (TIF) [file pone.0104215.s002.tif]
