# Supplementary material for: Effects of Aprepitant on the Pharmacokinetics of Controlled-Release Oral Oxycodone in Cancer Patients
Source: PLoS One. 2014 Aug 14;9(8):e104215. doi: 10.1371/journal.pone.0104215 (PMC4133207; doi:10.1371/journal.pone.0104215)
Supplement: Protocol S1 — Clinical Study Protocol (Japanese version). (PDF) [file pone.0104215.s004.pdf]

アプレピタント併用におけるオキシコドン徐放剤の薬物動態研究

Effect of Aprepitant on Pharmacokinecics of Controlled-Release Oxycodone

臨床試験実施計画書

研究責任者

南 博信　（神戸大学医学部附属病院腫瘍・血液内科教授）

神戸市中央区楠町７丁目５－１

TEL: 078-382-5820　FAX: 078-382-5821

研究事務局

藤原 豊　（神戸大学医学部附属病院腫瘍・血液内科助教）

神戸市中央区楠町７丁目５－１

TEL: 078-382-5820　FAX: 078-382-5821

目次

ページ
0. 概要 4
　0.1 目的 4
　0.2 対象 4
　0.3 本試験の意義 4
　0.4 患者登録基準 4
　0.5 方法 5
　0.6 評価項目 5
　0.7 研究機関 6
　0.8 患者登録機関 6
　0.10 研究事務局 6
1. 本試験の目的 7
2. 対象 7
3. 背景 7
4. 本試験の意義 8
5. 患者登録基準選択 8
　5.1 適格基準 8
　5.2 除外基準 8
6. エンドポイント 8
7. 研究方法 9
　7.1 シェーマ 9
　7.2 併用療法、支持療法 9
　7.3 薬物動態測定 10
　7.4 DNA 保存検体の採取 10
8. 評価項目 10
　8.1 患者評価項目 10
　8.2 DNA 保存用検体 11
9. エンドポイントに対する統計学的考察 11
　9.1 登録症例設定根拠 11
　9.2 薬物動態採血ポイント設定根拠 11
10. 研究機関 12
11. 患者登録期間 12
12. 公開データベース及び ID 12
13. 試験の安全性の確保 12

13.1　　被験者の安全性を確保するための基本的事項 12
13.2　　予想される有害事象 12
14. 倫理的事項 12
14.1　　患者の保護 13
14.2　　説明同意 13
14.3　　研究結果の開示 14
14.4　　研究計画書の開示 14
14.5　　検体の保存・管理 14
14.6　　個人識別情報の管理 14
14.7　　施設内倫理委員会などでの承認 14
15. 研究結果の公表 14
16. 研究費 14
17. 健康被害に関する補償 14
18. 知的財産権の帰属 14
19. 研究責任者、研究分担者、個人情報分担管理者 15
20. 研究事務局 15
21. 参考文献 15

0. 概要

0.1. 目的

アプレピタントによるオキシコドン及び代謝物の薬物動態への影響を検討する。

0.2. 対象

癌性疼痛に対してオキシコドン徐放剤（オキシコンチン®）を定期的に内服し、悪性腫瘍に対する抗悪性腫瘍薬投与の悪心、嘔吐の予防のためアプレピタント（イメンド®）投与を行う患者

0.3. 本試験の意義

CYP3A で代謝されるオキシコドンと CYP3A4 の軽度阻害剤であるアプレピタントの薬物相互作用を解析することで、オキシコドン徐放剤（オキシコンチン®）とアプレピタントを併用する際の適切な投与法を検討する。

0.4. 患者登録基準

0.4.1. 適格基準

① 悪性腫瘍と診断された患者
② 18 歳以上の患者
③ 抗悪性腫瘍薬投与時に制吐剤としてアプレピタント併用投与を行う患者
④ 癌性疼痛に対してオキシコドン徐放剤（オキシコンチン®）の定期内服（同一投与量を 1 日 2 回投与または 3 回投与）を 3 日以上している患者
⑤ 適切な主要臓器機能を有する患者
　肝機能　ＡＳＴ、ＡＬＴ：施設上限値の 2.5 倍以内
　　　　　総ビリルビン値：施設上限値の 1.5 倍以内
　腎機能　血清クレアチニン：施設上限値の 1.5 倍以内
⑥ 本試験への参加について文書による同意が得られた患者

0.4.2. 除外基準

①薬物動態採血期間（３日間）にオキシコドン速放剤（オキノーム®）の投与を必要とする患者（モルヒネ徐放剤（オプソ®）に変更可能な患者は除外しない）
②CYP3A4 の強い誘導剤および阻害剤、グレープフルーツジュース、セントジョーンズワートを併用している患者
③消化器系の機能障害またはオキシコドンやアプレピタントの吸収に著名な影響を及ぼす可能性がある消化器疾患を有する患者
④その他、試験責任医師または試験分担医師が本試験の対象として適当でないと判断した患者

0.5. 方法

実地医療においてアプレピタントを併用する抗悪性腫瘍薬投与をする患者において、アプレピタント投与前後でのオキシコドン及びその代謝物の薬物動態を比較検討する。

0.5.1　シェーマ
アプレピタント（イメンド®）内服時間は8：00
オキシコドン徐放剤（オキシコンチン®）内服時間は（アプレピタント無しの薬物動態採血１日前から）8：00、20：00の１日２回または8：00、16：00、22：00～24：00の１日３回とする

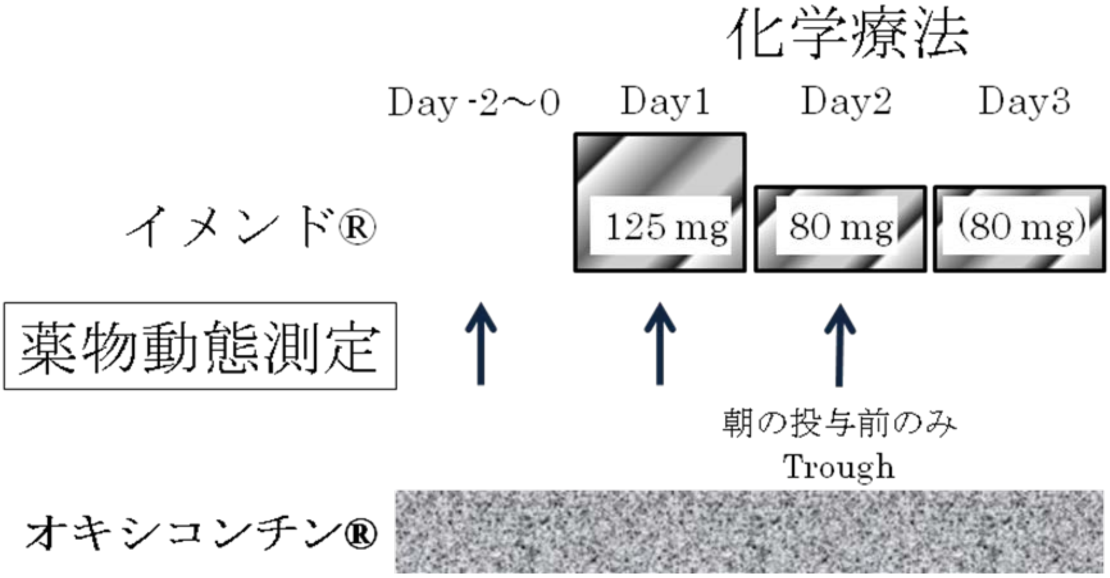

0.6.　評価項目
0.6.1. Endpoint
　Primary endpoint：アプレピタント投与前、投与１日目におけるオキシコドン及び代謝物の薬物動態
　Secondary endpoints：安全性、有害事象（悪心、嘔吐、便秘、傾眠傾向など）、有効性（制吐、鎮痛効果）、CYP3A4、CYP2D6の遺伝子多型とオキシコドン及び代謝物の薬物動態
0.6.2. 患者評価項目
１）患者背景：性別、登録時年齢、生年月日、患者識別番号
２）一般所見：PS、身長、体重
３）腫瘍所見：癌腫、病理学的・細胞診学的確定診断、病期（TNM分類）
４）血液学的検査所見：TP、Alb、T-Bil、AST、ALT、LDH、BUN、Cr
５）オキシコンチン®、オキノーム®、イメンド®内服状況（内服時間）
６）併用薬
0.6.3.　DNA保存用検体
血液より抽出したDNAサンプルは、CYP3A4、CYP2D6などの既知のオキシコドンの代謝に関連する遺伝子の多型、変異を検出するためにのみ用いる。

0.7.　研究機関

神戸大学医学部附属病院腫瘍・血液内科、呼吸器内科

0.8.　患者登録期間

神戸大学医学研究科長承認年月日から 2012 年 7 月 31 日まで 2 年間

0.9. 研究事務局

神戸大学医学部附属病院腫瘍・血液内科助教　藤原　豊
神戸市中央区楠町 7 丁目５－１
TEL:　078-382-5820　　FAX:　078-382-5821

「試験スケジュール」

オキシコドン徐放剤内服時間確認（アプレピタント無しの薬物動態採血１日前から）8：00、20：00 の 1 日 2 回または 8：00、16：00、22：00～24：00 の 1 日 3 回とする。

患者評価項目

１）患者背景：性別、登録時年齢、生年月日、患者識別番号
２）一般所見：PS、身長、体重
３）腫瘍所見：癌腫、病理学的・細胞診学的確定診断、病期（TNM 分類）
４）血液学的検査所見：TP、Alb、T-Bil、AST、ALT、LDH、BUN、Cr
５）オキシコンチン®、オキノーム®、イメンド®内服状況（内服時間）
６）併用薬

DNA 保存用検体採血

オキシコドン徐放剤単独投与時

オキシコンチン®	内服					
PK 採血	内服前<br>8：00	1 hr<br>9：00	2 hr<br>10：00	3 hr<br>11：00	5 hr<br>13：00	8 hr<br>16：00

イメンド、オキシコドン徐放剤同時併用時（Day1）

イメンド® 125mg	内服					
オキシコンチン®	内服					
PK 採血	内服前<br>8：00	1 hr<br>9：00	2 hr<br>10：00	3 hr<br>11：00	5 hr<br>13：00	8 hr<br>16：00

イメンド、オキシコドン徐放剤同時併用時（Day2）

イメンド® 80mg	内服	
オキシコンチン®	内服	
PK 採血	内服前<br>8：00	

1. 本試験の目的

アプレピタントによるオキシコドン及び代謝物の薬物動態への影響を検討する。

2. 対象

癌性疼痛に対してオキシコドン徐放剤（オキシコンチン®）を定期的に内服し、悪性腫瘍に対する抗悪性腫瘍薬投与の悪心、嘔吐の予防のためアプレピタント（イメンド®）投与を行う患者

3. 背景

がん性疼痛はがん患者の診断から死亡に至るまでのどの病期においても出現し、末期状態では約 70%のがん患者にて出現しているとの報告もある。1986 年 WHO はがん性疼痛治療に関するレポート「Cancer Pain Relief」を報告し、そこで提唱された３段階ラダーにおいて第３段階(高度)の疼痛に対しては強オピオイドであるモルヒネやオキシコドンが用いることが推奨されている[1]。オキシコドンの化学構造は、モルヒネあるいはコデインと類似するが、オキシコドンの構造には初回通過効果（First Pass Effect）を受けにくい特徴を有するため、モルヒネは経口投与すると速やかに代謝され、生物学的利用率は約 20～30%と低いのに比べて、オキシコドンの生物学的利用率は約 60～90%であり、臨床で使用される各種オピオイド作動薬の中で最も高い。オキシコドンは肝臓のチトクローム P450(CYP)2D6 でオキシモルフォン、CYP3A4 でノルオキシコドンに代謝され[2,3]、このうちオキシモルフォンは活性代謝産物であるが、ごく微量であり体への影響はほとんどみられないことが知られている。オキシコドンの鎮痛作用、副作用は主として未変化体が関与しているため、これらの代謝酵素の阻害は副作用の遷延を起こす可能性がある。

アプレピタント（イメンド®）は NK-1 受容体を選択的に競合拮抗する制吐剤である。無作為化比較試験において 5HT3 受容体拮抗薬およびデキサメサゾンと併用することで、がん化学療法に伴う急性期嘔吐、遅発性嘔吐の軽減が証明され[4-6]、米国臨床腫瘍学会のガイドラインにおいても高度催吐リスクの化学療法時には予防的に併用することが推奨されている[7]。しかしながらアプレピタントは CYP3A4 に軽度阻害作用を有することが知られ、併用するデキサメサゾンのクリアランスをアプレピタント 40 mg と 125 mg で、各々24.7 %, 47.5 %低下させるとの報告があり[8]、デキサメサゾンはアプレピタント併用時には、併用しない時に比べて減量して用いられている。

オキシコドンとアプレピタントを併用した場合、アプレピタントの CYP3A4 阻害作用にてオキシコドンの血中濃度が増加する可能性があり、オキシコドンの副作用（吐き気、便秘、眠気、譫妄など）が増加する可能性がある。しかしなが

ら今までにこれらの薬剤の相互作用を検討した報告はなく、オキシコドンの薬物動態の変化、有害事象の変化については不明である。

4. 本試験の意義

CYP3A で代謝されるオキシコドンと CYP3A4 の軽度阻害剤であるアプレピタントの薬物相互作用を解析することで、オキシコドン徐放剤（オキシコンチン®）とアプレピタントを併用する際の適切な投与法を検討する。これらの研究結果によりオキシコドンの有害事象の軽減に貢献ができる可能性がある。

5. 患者登録基準

適格基準のすべてを満たし、除外基準のいずれにも該当しない患者を適格とする。

5.1. 適格基準

① 悪性腫瘍と診断された患者
② 18 歳以上の患者
③ 抗悪性腫瘍薬投与時に制吐剤としてアプレピタント併用投与を行う患者
④ 癌性疼痛に対してオキシコドン徐放剤（オキシコンチン®）の定期内服（同一投与量を 1 日 2 回投与または 3 回投与）を 3 日以上している患者
⑤ 適切な主要臓器機能を有する患者
　肝機能　ＡＳＴ、ＡＬＴ：施設上限値の 2.5 倍以内
　　　　　総ビリルビン値：施設上限値の 1.5 倍以内
　腎機能　血清クレアチニン：施設上限値の 1.5 倍以内
⑥ 本試験への参加について文書による同意が得られた患者

5.2. 除外基準

① 薬物動態採血期間（３日間）にオキシコドン速放剤（オキノーム®）の投与を必要とする患者（モルヒネ徐放剤（オプソ®）に変更可能な患者は除外しない）
② CYP3A4 の強い誘導剤および阻害剤、グレープフルーツジュース、セントジョーンズワートを併用している患者
③ 消化器系の機能障害またはオキシコドンやアプレピタントの吸収に著名な影響を及ぼす可能性がある消化器疾患を有する患者（活動性潰瘍性病変、コントロール不良な悪心、嘔吐、下痢、吸収不良症候群、小腸切除など）
④ その他、試験責任医師または試験分担医師が本試験の対象として適当でないと判断した患者

6. エンドポイント

Primary endpoint：アプレピタント投与前、投与１日目におけるオキシコド

ン及び代謝物の薬物動態
　Secondary endpoints：安全性、有害事象（悪心、嘔吐、便秘、傾眠傾向など）、有効性（制吐、鎮痛効果）、CYP3A4、CYP2D6 の遺伝子多型とオキシコドン及び代謝物の薬物動態

7. 研究方法

実地医療においてアプレピタントを併用する抗悪性腫瘍薬投与をする患者において、アプレピタント投与前後でのオキシコドン及びその代謝物の薬物動態を比較検討する。

7.1.　シェーマ

アプレピタント内服時間は 8：00
オキシコンチン内服時間は（アプレピタント無しの薬物動態採血１日前から）8：00、20：00 の 1 日 2 回または 8：00、16：00、22：00～24：00 の 1 日 3 回とする。

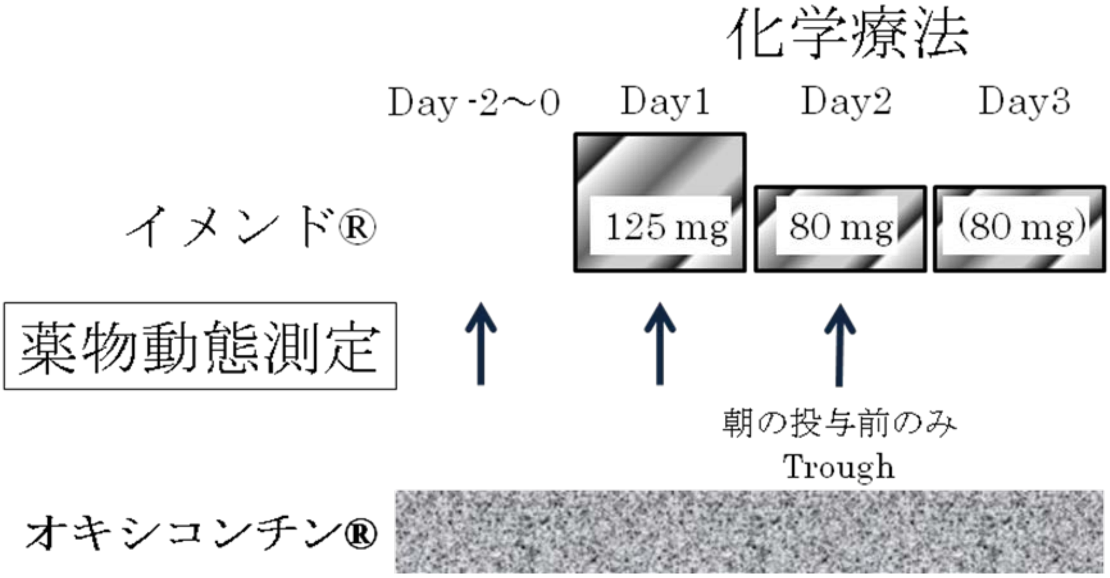

7.2.　併用療法、支持療法

原疾患、抗悪性腫瘍療法に対する支持療法は必要に応じて行う。好中球減少時の G-CSF、輸血（赤血球、血小板）においては適切な臨床判断のもと行うことを許容する。ただし CYP3A4 の強い誘導剤はイメンド投与前 14 日～Day３の併用を CYP3A4 の強い阻害剤はイメンド投与日～Day3 の併用は可能な限り避ける。また試験期間中はグレープフルーツおよびグレープフルーツジュースの摂取は禁止とする。

7.3.　薬物動態測定

１）アプレピタント投与前、投与１日目に対象患者より血漿採取のために各 4ml の血液を次のポイントで採取する。

採血ポイント

・オキシコンチン単独投与時（イメンドの内服が無い時）

・オキシコンチン、イメンド同時併用時（イメンドの内服が有る時）

採血ポイント：内服直前, 投与 1h, 2h, 3h, 5h, 8h

・イメンド投与 2 日目：内服直前

採血回数は合計 13 回、1 回の採血は 4ml で合計 52ml

遠心分離器で採血後速やかに、1500g で 10 分間遠心をする

２）各医療機関において血漿分離を行い検体保存用チューブに 0.5ml ずつ 3 本に分注し、-80 度の Deep Freezer に保存する。

7.4.　DNA 保存用検体の採取

7.4.1.　末梢血単核球の採取

末梢血（全血で約10ml）を採取する。採血後すみやかに単核球を分離し、凍結保存する。

7.4.2.　DNA保存検体の廃棄方法

血液から抽出したDNAサンプルは保存され、本試験における解析に用いられる。解析後の残余試料は本試験が完了し結果が報告された時点で、ほかのすべての残余試料とともに熱処理など適切な方法で廃棄される。

7.4.3.　検体の保管

各医療機関において-80 度の Deep freezer にて、測定までの間保存を行う。

7.4.4.　検体の測定

神戸大学医学部附属病院腫瘍・血液内科および外部検査機関において行う。測定項目については、**8.評価項目**に記載する

7.4.5.　検体の匿名化

検体は登録された時点で、個人情報管理者の管理の下で管理され、匿名化を行う。具体的には、個人情報の管理は個人情報管理者により、個人情報管理専用のコンピュータによりパスワード、暗号化を用いて管理され、個人情報および症例・符号対照表には個人情報管理者または個人情報分担管理者以外のアクセスができないようにする。匿名化については症例登録時に検体に対応する個人識別情報は検体組織部位、症例番号、採取時期を示す 6 文字の英数字に変換し、以後、検体は符号化された番号のみで取り扱い、解析に用いる。

8. 評価項目

8.1. 患者評価項目

１）患者背景：性別、登録時年齢、生年月日、患者識別番号
２）一般所見：PS、身長、体重
３）腫瘍所見：癌腫、病理学的・細胞診学的確定診断、病期（TNM 分類）
４）血液学的検査所見：TP、Alb、T-Bil、AST、ALT、LDH、BUN、Cr
５）オキシコンチン®、オキノーム®、イメンド®内服状況（内服時間）
６）併用薬

8.2. DNA 保存用検体

血液より抽出したDNAサンプルは、CYP3A4、CYP2D6などの既知のオキシコドンの代謝に関連する遺伝子の多型、変異を検出するためにのみ用いる。将来オキシコドン、アプレピタントの代謝に関連する遺伝子が新たに同定された場合には再解析を行うことにする。この場合には再度倫理委員会に申請を行い、承認を必要とする。ただし被験者に対する再同意は必要としない。また本試験の結果によって遺伝子解析の実施の必要がないと判断された場合には、解析を実施しないこともある。

9. エンドポイントに対する統計学的考察

9.1. 登録症例設定根拠

医薬品インタビューフォームにおいて癌疼痛患者における定常状態での体内動態パラメータ(n=32)より、オキシコドン AUC は 216.2±97.4(ng.hr/ml)でありCV は 45.1%であった。アプレピタント投与前後での AUC が 40%変化すれば有意であると仮定して、α=0.05(片側検定)、β=0.2 のもとで症例数は 17 例が必要とされる。脱落例も入れて、20 例を目標症例数とした。

	投与量 (mg)	Tmax (hr)	投与量換算値(20mg/回に換算)			Ctrough 比*	AUC 比*
			Cmax (ng/mL)	Ctrough (ng/mL)	AUC (ng/hr/mL)		
平均値	21.3	2.8	47.5	24.6	216.2	1.05	0.86
標準偏差	18.4	1.8	19.7	13.0	97.4	0.91	1.04
最小値	10	0.8	3.5	1.7	15.0	0.16	0.14
最大値	100	5.8	96.0	58.5	467.0	4.98	6.29

*オキシコドンとノルオキシコドンの Ctrough、AUC の比

9.2. 薬物動態採血ポイント設定根拠

オキシコドン徐放剤（オキシコンチン®）の薬物動態（**9.1.参照**）から半減期を求めるためには、終末相における薬物動態採血が数ポイント必要となる。本試験において、1 日 3 回投与（8 時間ごと）の被験者では投与後 8 時間までの採血

が限界となる。この場合、正確な半減期は算出不能と考えるが、$AUC_{0\text{-}8h}$の薬物動態をPrimary endpointとして被験者内の薬物動態を解析することは可能と考えられる。

10. 研究機関

神戸大学医学部附属病院腫瘍・血液内科、呼吸器内科

11. 患者登録期間

神戸大学医学研究科長承認年月日から2012年7月31日まで2年間

12. 公開データベース及びID

UMIN試験ID：UMIN000003580
受付番号：R000004314
試験名： アプレピタント併用におけるオキシコドン徐放剤の薬物動態研究
登録日：2010年6月1日
利用者名：藤原豊

13. 試験の安全性の確保

13.1. 被験者の安全性を確保するための基本的事項

試験責任（担当）医師は、被験者の試験参加中、必要かつ適切な観察・検査を行い、被験者の安全性確保に留意する。有害事象の発現に際しては、必要に応じて適切な処置を施し、被験者の安全性確保に留意するとともに、その原因究明に努める。

13.2. 予想される有害事象（オキシコンチン®添付文書より）

（１）臨床上重要と考えられる副作用の発現頻度
承認時における安全性評価対象例302例中，副作用は231例（76.5%）に認められた。主なものは眠気160例(53.0%),便秘116例(38.4%),嘔気116例(38.4%),嘔吐56例（18.5%），食欲不振12例（4.0%），眩暈10例（3.3%），そう痒感10例（3.3%）等であった。
（２）重大な副作用
1)ショック、アナフィラキシー様症状（頻度不明）
2)依存性（頻度不明）
3)呼吸抑制（頻度不明）
4)錯乱、譫妄（頻度不明）
5)無気肺、気管支痙攣、喉頭浮腫（頻度不明）
6)麻痺性イレウス（0.1～1%未満）、中毒性巨大結腸（頻度不明）

7)肝機能障害（頻度不明）

14. 倫理的事項

14.1. 患者の保護

本試験を実施するにあたり、「ヘルシンキ宣言」(2002 年 米国ワシントン）および「臨床研究に関する倫理指針」(2008 年 7 月 31 日 厚生労働省）の倫理的原則を遵守して、患者の人権、福祉および安全を最大限に確保することとする。本試験に関する、有害事象、研究結果その他関連データを報告する場合には、患者の身元の秘密を保全し、人権保護について十分配慮することとする。

14.2. 説明同意

14.2.1. 患者への説明

担当医は、登録前に同意説明文書・同意書に基づき、事前に本研究の意義、目的、方法、予測される結果や不利益について検体提供者に説明する。担当医並びに検体提供者は、同意書に署名及び日付（説明日）を記載する。同意説明文書・同意書（写）を検体提供者に渡すとともに、原本をカルテ内に保管する。

14.2.2. 同意説明文書・同意書による検体提供者への説明事項

同意取得に際し、下記説明事項を同意説明文書・同意書を用いて説明する。

- 研究概要
- 研究目的
- 研究方法
- 関連する遺伝子の変異解析について
- 参加予定期間
- 参加予定患者数
- 研究期間中及び終了後の試(資)料等の取り扱いの方針
- 予想される臨床上の利益(効果)及び不利益(副作用)
- 本試験の実施機関
- 健康被害が発生した場合に受けることができる治療
- 研究協力の任意性
- 同意撤回の自由
- 新しい重大な情報の開示
- 研究への参加が中止となる条件
- 個人へのプライバシーの保護
- 研究成果の公表
- 知的財産権の帰属
- 費用の負担

- 謝礼の有無
- 研究計画書等の開示
- 守らなければならない事項
- 研究責任者・研究協力者の氏名・職名・連絡先
- 相談窓口

14.2.3. 患者の同意
試験についての説明後、患者が試験の内容をよく理解したことを確認した上で、試験への参加について文書にて自由意思による検体提供者の同意を得る。本試験への参加については患者の完全な自由意思によるものとし、患者本人が試験参加に同意した場合、同意を得た日付を記載し患者本人および説明を行った医師が署名する。同意書は、1 部は患者本人に手渡し、1 部はカルテに保管する。

14.3. 研究結果の開示
本研究の研究結果により被検者の治療や医学上の利益に重大な影響を与えると判明した場合には、再度説明を行なう。

14.4. 研究計画書の開示
被検者もしくはその関係者が本研究の実施計画書の開示を希望される場合は、必要に応じて開示を行なう。

14.5. 検体の保存・管理
採取した検体は速やかに研究施設内の冷凍庫に保存され、厳重に管理される。

14.6. 個人識別情報の管理
提供を受けた検体は、個人情報管理者が検体識別番号により符号化する。提供者と符号の対照表は個人情報管理者により厳重に保管される。

14.7. 施設内倫理審査委員会などでの承認
本試験は神戸大学医学部倫理委員会において承認を受けている。

15. 研究結果の公表
研究代表者が共同研究者と協議の上、研究代表者、共同研究者、または研究協力者が論文、学会発表を行う。

16. 研究費
一般診療および通常臨床検査に関わる費用は通常の医療保険制度に沿った患者負担とする。薬物動態解析及び DNA 検体に関する費用に関しては神戸大学医学部腫瘍・血液内科において負担する。

17. 健康被害に関する補償

本試験に起因した健康被害に生じた場合には、通常の保険医療に基づいた適切な医療を提供する。補償は医師に過失がない限り行わない。

18.　知的財産権の帰属

本研究から生じる知的財産権は神戸大学または研究者に帰属する。

19. 研究責任者、研究分担者、個人情報分担管理者

研究責任者：南 博信　（神戸大学医学部附属病院腫瘍・血液内科教授）
神戸市中央区楠町７丁目５－１
TEL: 078-382-5820　FAX: 078-382-5821

研究分担者：向原 徹　（神戸大学医学部附属病院腫瘍・血液内科准教授）
松岡 宏　（神戸大学医学部附属病院腫瘍・血液内科准教授）
岡村 篤夫　（神戸大学医学部附属病院腫瘍・血液内科助教）
薬師神 公和　（神戸大学医学部附属病院腫瘍・血液内科助教）
清田 尚臣　（神戸大学医学部附属病院腫瘍・血液内科助教）
藤原 豊　（神戸大学医学部附属病院腫瘍・血液内科助教）
茶屋原 菜穂子（神戸大学医学部附属病院腫瘍・血液内科医員）
豊田 昌徳　（神戸大学医学部附属病院腫瘍・血液内科医員）
島田 貴則　（神戸大学医学部附属病院腫瘍・血液内科医員）
富岡 秀夫　（神戸大学医学部附属病院腫瘍・血液内科医員）
船越 洋平　（神戸大学医学部附属病院腫瘍・血液内科医員）
乾 由美子　（神戸大学医学部附属病院腫瘍・血液内科医員）
西村 義博　（神戸大学医学部附属病院呼吸器内科准教授）
小谷 義一　（神戸大学医学部附属病院呼吸器内科講師）
船田 泰弘　（神戸大学医学部附属病院呼吸器内科助教）
中田 恭介　（神戸大学医学部附属病院呼吸器内科医員）
櫨木 暢子　（神戸大学医学部附属病院呼吸器内科医員）
冨田 菜々子　（神戸大学医学部附属病院呼吸器内科医員）

個人情報管理者　：
前田英一（神戸大学医学部附属病院医療情報部特命教授）

個人情報分担管理補助者：
若宮浩子（神戸大学医学部附属病院腫瘍・血液内科技術補佐員）

20. 研究事務局

神戸大学医学部附属病院腫瘍・血液内科助教　藤原　豊
神戸市中央区楠町７丁目５－１

TEL： 078-382-5820　 FAX： 078-382-5821

21. 参考文献

1. Azevedo Sao Leao Ferreira K, Kimura M, Jacobsen Teixeira M. The WHO analgesic ladder for cancer pain control, twenty years of use. How much pain relief does one get from using it? Support Care Cancer 2006;14:1086-93.
2. Lugo RA, Kern SE. The pharmacokinetics of oxycodone. J Pain Palliat Care Pharmacother 2004;18:17-30.
3. Lalovic B, Kharasch E, Hoffer C, Risler L, Liu-Chen LY, Shen DD. Pharmacokinetics and pharmacodynamics of oral oxycodone in healthy human subjects: role of circulating active metabolites. Clin Pharmacol Ther 2006;79:461-79.
4. Hesketh PJ, Grunberg SM, Gralla RJ, et al. The oral neurokinin-1 antagonist aprepitant for the prevention of chemotherapy-induced nausea and vomiting: a multinational, randomized, double-blind, placebo-controlled trial in patients receiving high-dose cisplatin--the Aprepitant Protocol 052 Study Group. J Clin Oncol 2003;21:4112-9.
5. Poli-Bigelli S, Rodrigues-Pereira J, Carides AD, et al. Addition of the neurokinin 1 receptor antagonist aprepitant to standard antiemetic therapy improves control of chemotherapy-induced nausea and vomiting. Results from a randomized, double-blind, placebo-controlled trial in Latin America. Cancer 2003;97:3090-8.
6. Warr DG, Grunberg SM, Gralla RJ, et al. The oral NK(1) antagonist aprepitant for the prevention of acute and delayed chemotherapy-induced nausea and vomiting: Pooled data from 2 randomised, double-blind, placebo controlled trials. Eur J Cancer 2005;41:1278-85.
7. Kris MG, Hesketh PJ, Somerfield MR, et al. American Society of Clinical Oncology guideline for antiemetics in oncology: update 2006. J Clin Oncol 2006;24:2932-47.
8. Nakade S, Ohno T, Kitagawa J, et al. Population pharmacokinetics of aprepitant and dexamethasone in the prevention of chemotherapy-induced nausea and vomiting. Cancer Chemother Pharmacol 2008;63:75-83.
