# Supplementary material for: Effects of Aprepitant on the Pharmacokinetics of Controlled-Release Oral Oxycodone in Cancer Patients
Source: PLoS One. 2014 Aug 14;9(8):e104215. doi: 10.1371/journal.pone.0104215 (PMC4133207; doi:10.1371/journal.pone.0104215)
Supplement: Protocol S2 — Clinical Study Protocol (English version). (DOCX) [file pone.0104215.s005.docx]

**Effect of Aprepitant on the Pharmacokinetics of Controlled-release Oxycodone**

**Clinical Study Protocol**

**Principle investigator: Prof. Hironobu Minami**

Division of Medical Oncology/Hematology, Kobe University Graduate School of Medicine

7-5-2 Kusunoki-cho, Chuo-ku, Kobe, 650-0017, JAPAN

Phone: +81-78-382-5820　　　FAX: +81-78-382-5821

E-mail: hminami@med.kobe-u.ac.jp

**Research secretariat: Yutaka Fujiwara**

Division of Medical Oncology/Hematology, Kobe University Graduate School of Medicine

**1. Study Purpose**

To investigate the effect of aprepitant on the pharmacokinetics of oxycodone and its metabolites.

**2. Subjects**

Subjects in this study were malignant solid tumor patients with regular oral intake of controlled-release (CR) oxycodone for cancer pain and were planned to receive chemotherapy with aprepitant for chemotherapy-induced nausea and vomiting (CINV).

**3. Background**

The WHO analgesic ladder recommends the use of opioid such as morphine and oxycodone for cancer pain control ^1^. The chemical structure of oxycodone is similar to that of codeine or morphine. Morphine is rapidly metabolized after oral administration, with its bioavailability being as low as approximately 20% to 30%. In contrast, oxycodone has a structure that is less susceptible to the first pass effect and a bioavailability of approximately 60% to 90%, which is the highest of the clinically used opioid agonists. In the liver, oxycodone is metabolized to oxymorphone by cytochrome P450(CYP)2D6 and to noroxycodone by CYP3A4 ^2,3^. Although oxymorphone is an active metabolite, its plasma concentration is very low and its effect on the body is minor. Because the analgesic effect and side effects of oxycodone primarily involve the unchanged form, inhibition of metabolic enzymes might cause the prolongation of side effects.

Aprepitant is an antiemetic drug that selectively and competitively antagonizes the NK-1 receptor. Randomized controlled trials demonstrated that combination of aprepitant with 5HT_3_ receptor antagonists and dexamethasone ameliorated CINV in the acute and delayed phase ^4-6^. The guidelines of the American Society of Clinical Oncology recommend the prophylactic use of aprepitant in combination with 5HT3 receptor antagonists and dexamethasone in chemotherapy for highly emetic risk ^7^. However, aprepitant has a mild inhibitory effect on CYP3A4, with 40 or 125 mg of aprepitant being reported to reduce clearance of combined dexamethasone by 24.7% or 47.5%, respectively.^8^ Therefore, when aprepitant is used in combination, the dose of dexamethasone is reduced compared to when aprepitant is not used.

Similarly, in combination use of aprepitant with oxycodone, the inhibitory effect on CYP3A4 of aprepitant might result in an increase in the plasma concentration of oxycodone, and accordingly an increase in the side effects of oxycodone (nausea, constipation, lethargy, delirium, and others). To our knowledge, however, the interaction of these two drugs has not been investigated, and the effect of aprepitant on the pharmacokinetics and side effects of oxycodone and its metabolites remain to be elucidated.

**4. Significance of the study**

We have investigated the possible effects of the mild CYP3A4 inhibitor aprepitant on the pharmacokinetics of orally administered CR oxycodone in patients with cancer pain. The results of this study will contribute to an appropriate administration method for the combination use of aprepitant and CR oxycodone.

**5. Criteria for subject enrollment**

**5.1. Eligibility criteria**

1) Patients histologically diagnosed with a malignant solid tumor.

2) Patients 18 years of age or older.

3) Patients administered aprepitant in combination with chemotherapy as clinical practice.

4) Patients with regular intake (twice or three times daily with the same dose) of CR oxycodone for cancer pain for more than three days.

5) Patients with adequate organ function as follows:

Liver function: AST and ALT less than 2.5 times of ULN.

Total bilirubin value less than 1.5 times of ULN.

Renal function: Serum creatinine level less than 1.5 times of ULN.

6) Patients from whom written informed consent for participation in the study is obtained.

**5.2. Exclusion criteria**

1) Patients in need of fast-release oxycodone administration during the blood sampling period for pharmacokinetics.

2) Patients taking strong CYP3A4 inducers or inhibitors, grapefruit juice, or St. John's Wort.

3) Patients with gastrointestinal impairment that might substantially affect the absorption of aprepitant or oxycodone.

4) In addition, patients who the investigator determines to be unsuitable for the study.

**6. Endpoint**

Primary endpoint: pharmacokinetics of oxycodone and its metabolites before and on Day 1 of aprepitant administration.

Secondary endpoints: safety, adverse events (nausea, vomiting, constipation, lethargy, and others), effectiveness (antiemetic effect, analgesic effect), and pharmacokinetics of oxycodone and its metabolites on genetic polymorphisms of CYP2D6 and CYP3A4.

**7. Study methods**

The pharmacokinetics of oxycodone and its metabolites before and after aprepitant administration will be investigated and compared.

**7.1. Schema in oral administration of aprepitant and CR ocycodone**

1) Oral administration of aprepitant at 8:00AM.

2) Oral administration of CR oxycodone twice daily at 8:00AM and 8:00PM, or three times daily at 8:00AM, 4:00PM, and from 12:00PM.

| **CR Oxycodone alone (Day1）** | | | | | | |
| --- | --- | --- | --- | --- | --- | --- |
| CR Oxycodone | ● |  |  |  |  |  |
| PK sampling | Before  8：00 | 1 hr  9：00 | 2 hr  10：00 | 3 hr  11：00 | 5 hr  13：00 | 8 hr  16：00 |
| **CR Oxycodone with Aprepitant（Day2）** | | | | | | |
| Aprepitant 125mg | ● |  |  |  |  |  |
| CR Oxycodone | ● |  |  |  |  |  |
| PK sampling | Before  8：00 | 1 hr  9：00 | 2 hr  10：00 | 3 hr  11：00 | 5 hr  13：00 | 8 hr  16：00 |
| **CR Oxycodone with Aprepitant（Day3）** | | | | | | |
| Aprepitant 80mg | ● |  | | | | |
| CR Oxycodone | ● |  | | | | |
| PK sampling | Before  8：00 |  | | | | |

**7.2. Combination therapy and supportive care**

Supportive care for primary disease or antineoplastic therapy will be carried out as necessary.

**7.3. Pharmacokinetics measurement**

1) 4 ml of blood will be collected to obtain plasma samples at the following time points:

・Day 1: CR oxycodone is administered alone (with no administration of aprepitant).

・Day 2: CR oxycodone and aprepitant are simultaneously administered (with oral administration of aprepitant).

Blood collection points: immediately before and 1 h-, 2 h-, 3 h-, 5 h-, and 8 h-post administration of CR oxycodone at 8:00AM.

・ Day 3: aprepitant administration: immediately before administration of CR oxycodone at 8:00AM.

4 ml of blood will be collected 13 times and a total of 52 ml will be sampled.

2) After blood was collected in lithium heparin-containing tubes, plasma was separated within 30 min by centrifugation at 1,500×g for 10 min at 4 °C and stored at -80 °C until analysis.

**7.4. Collection of DNA for storage**

**7.4.1. Collection of peripheral blood mononuclear cells**

Peripheral blood will be collected (approximately 10 ml of whole blood). After collection, mononuclear cells will be separated and immediately frozen for storage.

**7.4.2. Disposal of DNA samples**

DNA samples extracted from blood will be stored and used for analysis. Residual samples, along with all the other remaining samples, will be discarded at the time results are reported.

**7.4.3.** **Anonymity of DNA samples**

Samples will be managed and anonymized at the time of registration by the personal data manager. The personal data and case-coding correspondence table will be stored to ensure that only the personal data manager can access them.

**8. Evaluation items**

**8.1. Items for patient assessment**

1) Patient characteristics: gender, age at time of registration, date of birth, and patient identification number.

2) General findings: Performance status, height, and weight.

3) Tumor findings: cancer type, pathological diagnosis, and clinical stage (TNM classification).

4) Hematology findings: TP, Alb, T-Bil, AST, ALT, LDH, BUN, and Cr.

5) Oral administration time for CR oxycodone and aprepitant.

6) Concomitant medications.

**8.2. DNA samples for storage**

DNA samples extracted from blood will be used only for detecting mutations and polymorphisms in known gene such as CYP3A4 and CYP2D6 that are involved in the metabolism of oxycodone.

**9. Statistical considerations for the end point analysis**

**9.1. Basis for registration of cases**

This study was designed in order to exclude a clinically significant higher exposure to oxycodone and its metabolites. The primary hypothesis was that coadministration of aprepitant would not increase the plasma concentration of oxycodone to a clinically meaningful degree, i.e., the ratio of the geometric mean AUC_0→8_ for oxycodone between period A and period B would be < 1.33. Package insert of oxycodone reports that the AUC of oxycodone in steady state was 216.2 ± 97.4 ng.hr/ml [mean ± standard deviation, coefficient of variance (CV) was 45.1%] in patients with cancer pain (n=32). We estimated that 20 subjects were needed to detect a 33% difference in the AUC_0→8_ for oxycodone at a power of 80% and level of significance p < 0.025 (one-sided).

|  | Dose  (mg) | T_max_  (hr) | Corrected PK parameters of oxycodone | | | C_trough_ratio^*^ | AUCratio^**^ |
| --- | --- | --- | --- | --- | --- | --- | --- |
|  |  |  | C_max_  (ng/ml) | C_trough_  (ng/ml) | AUC  (ng/hr/ml) |  |  |
| mean | 21.3 | 2.8 | 47.5 | 24.6 | 216.2 | 1.05 | 0.86 |
| standarddeviation | 18.4 | 1.8 | 19.7 | 13.0 | 97.4 | 0.91 | 1.04 |
| minimum values | 10 | 0.8 | 3.5 | 1.7 | 15.0 | 0.16 | 0.14 |
| maximum values | 100 | 5.8 | 96.0 | 58.5 | 467.0 | 4.98 | 6.29 |

^*^C_trough_ ratio of oxycodone to noroxycodone

^**^AUC ratio of oxycodone to noroxycodone

Values were corrected for dose, assuming that all patients received 20 mg of oxycodone

**10. Study Institution**

Division of Medical Oncology/Hematology, Kobe University Graduate School of Medicine

**11. Study period**

From June 1st, 2010 to July 31th, 2012.

**12. Trial registration**

Trial registration ID：UMIN000003580

[Official scientific title of the study](http://www.umin.ac.jp/ctr/UMIN-CTR_Yougo.htm#titlej): Effect of Aprepitant on Pharmacokinecics of Controlled-Release Oxycodone

Date of registration: June 1st, 2010

User name: Yutaka Fujiwara

**13. Ensuring study safety**

**13.1. Basic precautions to ensure safety of subjects**

During the study, the study director (attendant physician) must appropriately observe the subjects when necessary, and note their safety. Upon signs of adverse events, appropriate action should be taken as required to ensure the safety of subjects, and the study director must strive to find the cause.

**14. Ethical matters**

**14.1. Protection of subjects**

In carrying out the study, maximum protection of subjects’ human rights, welfare, and safety will be provided in compliance with the ethical principles of the "Declaration of Helsinki" and "Ethical Guidelines for Clinical Research" (Ministry of Health, Labor and Welfare, July 31, 2008). When adverse events, study results, or other relevant data of the study are disclosed, subjects’ personal information will be kept confidential and due consideration for protection of human rights will be given.

**14.2. Informed consent**

**14.2.1. Explanation to subjects**

The investigator is responsible for ensuring that the patient understands the potential risks and benefits of participating in the study, including answering any questions the patient may have throughout the study and sharing in a timely manner any new information that may be relevant to the patient’s willingness to continue his or her participation in the study in a timely manner.

The informed consent form will be used to explain the potential risks and benefits of study participation to the patient in simple terms before the patient is entered into the study and to document that the patient is satisfied with his or her understanding of the potential risks and benefits of participating in the study and desires to participate in the study.

**14.2.2. Informed consent document and form**

Prior to obtaining consent, the following matters will be explained with reference to the document for informed consent form:

- - - - Study overview
      - Study purpose
      - Study methods
      - The mutation analysis of related genes
      - Study period
      - Planned number of subjects
      - Policy for sample handling during and after the study period
      - Expected clinical benefits (effects) and disadvantages (side effects)
      - Implementing institution of the study
      - Therapies that can be received in occurrence of health hazards
      - Arbitrary of participation in the study
      - Freedom of consent withdrawal from study
      - Disclosure of new critical information
      - Conditions where participation in the study is discontinued
      - Personal information protection poricy
      - Publication of study results
      - Ownership of intellectual property rights
      - Allocation of expenses
      - Presence or absence of reward
      - Disclosure of study protocol and other materials
      - Compliance rules
      - Names, job titles, and contact information of the investigators
      - Inquiry counter

**14.2.3. Patient consent**

After explanation of the study, patients should be confirmed their full understanding of the study. Thereafter, all patients provided written informed consent after the physician and patient record the date and sign the informed consent form, the physician will hand over a copy of the consent form to the subject, and store the originals in the patient’s medical records.

**14.3. Disclosure of study results**

If this study has a serious effect on the treatment of subjects or on medical profits, we will again explain about study results.

**14.4. Disclosure of study protocol**

If a subject or his/her relatives wishes the study protocol to be disclosed, it will be disclosed as necessary.

**14.5. Storage and management of collected samples**

Collected samples will be stored immediately in a deep freezer at research facilities and managed appropriately.

**14.6. Management of personal identification information**

Samples will be managed and anonymized at the time of registration by the personal data manager. The personal data and case-coding correspondence table will be stored to ensure that only the personal data manager can access them.

**14.7. Approval by Institutional Review Board**

This study approval was obtained from the Institutional Review Board of Kobe University Hospital.

**15. Publication of study results**

Study results will be published or presented in academic conferences by study investigators.

**16. Research funding**

This study was supported by a grant for Research on Applying Health Technology from the Ministry of Health, Labour, and Welfare of Japan and Yokoyama-Rinsyo foundation.

**17. Ownership of intellectual property rights**

　Ownership of intellectual property rights attribute to Kobe University orinvestigators.

**18. Investigators**

- Principle investigator: Prof. Hironobu Minami

Division of Medical Oncology/Hematology, Kobe University Graduate School of Medicine

- Sub-investigators:

Toru Mukohara, Yutaka Fujiwara, Naomi Kiyota, Masanori Toyoda, Takanobu Shimada, and Yoshinori Imamura

- Personal data manager: Eiichi Maeda

Division of Medical Informatics and Bioinformatics, Kobe University Graduate School of Medicine

- Research secretariat: Yutaka Fujiwara

Division of Medical Oncology/Hematology, Kobe University Graduate School of Medicine

**19.　Reference**

1. Azevedo Sao Leao Ferreira K, Kimura M, Jacobsen Teixeira M. The WHO analgesic ladder for cancer pain control, twenty years of use. How much pain relief does one get from using it? Support Care Cancer 2006;14:1086-93.

2. Lugo RA, Kern SE. The pharmacokinetics of oxycodone. J Pain Palliat Care Pharmacother 2004;18:17-30.

3. Lalovic B, Kharasch E, Hoffer C, Risler L, Liu-Chen LY, Shen DD. Pharmacokinetics and pharmacodynamics of oral oxycodone in healthy human subjects: role of circulating active metabolites. Clin Pharmacol Ther 2006;79:461-79.

4. Hesketh PJ, Grunberg SM, Gralla RJ, et al. The oral neurokinin-1 antagonist aprepitant for the prevention of chemotherapy-induced nausea and vomiting: a multinational, randomized, double-blind, placebo-controlled trial in patients receiving high-dose cisplatin--the Aprepitant Protocol 052 Study Group. J Clin Oncol 2003;21:4112-9.

5. Poli-Bigelli S, Rodrigues-Pereira J, Carides AD, et al. Addition of the neurokinin 1 receptor antagonist aprepitant to standard antiemetic therapy improves control of chemotherapy-induced nausea and vomiting. Results from a randomized, double-blind, placebo-controlled trial in Latin America. Cancer 2003;97:3090-8.

6. Warr DG, Grunberg SM, Gralla RJ, et al. The oral NK(1) antagonist aprepitant for the prevention of acute and delayed chemotherapy-induced nausea and vomiting: Pooled data from 2 randomised, double-blind, placebo controlled trials. Eur J Cancer 2005;41:1278-85.

7. Kris MG, Hesketh PJ, Somerfield MR, et al. American Society of Clinical Oncology guideline for antiemetics in oncology: update 2006. J Clin Oncol 2006;24:2932-47.

8. Nakade S, Ohno T, Kitagawa J, et al. Population pharmacokinetics of aprepitant and dexamethasone in the prevention of chemotherapy-induced nausea and vomiting. Cancer Chemother Pharmacol 2008;63:75-83.
